# Engineering is evolution: a perspective on design processes to engineer biology

**DOI:** 10.1038/s41467-024-48000-1

**Published:** 2024-04-29

**Authors:** Simeon D. Castle, Michiel Stock, Thomas E. Gorochowski

**Affiliations:** 1 School of Biological Sciences, University of Bristol, Life Sciences Building, 24 Tyndall Avenue, Bristol, UK; 2 KERMIT, Department of Data Analysis and Mathematical Modelling, Ghent University, Ghent, Belgium; 3 BrisEngBio, School of Chemistry, University of Bristol, Cantock’s Close, Bristol, UK

**Keywords:** Biotechnology, Evolution, Engineering, Synthetic biology

## Abstract

Careful consideration of how we approach design is crucial to all areas of biotechnology. However, choosing or developing an effective design methodology is not always easy as biology, unlike most areas of engineering, is able to adapt and evolve. Here, we put forward that design and evolution follow a similar cyclic process and therefore all design methods, including traditional design, directed evolution, and even random trial and error, exist within an evolutionary design spectrum. This contrasts with conventional views that often place these methods at odds and provides a valuable framework for unifying engineering approaches for challenging biological design problems.

## Abstract (web summary)

Evolutionary and rational design approaches are commonly used to engineer biological systems but are typically seen at odds with each other. In this perspective the authors argue for the concept of an evolutionary design spectrum to help unify and compare these design methodologies to support more effective biological engineering.

## Introduction

Synthetic biology aims to apply engineering principles and the design process to create biological systems with new and desired functionalities^1^. While it is seen by many as a young field that promises a lot, we have, in fact, been making with biology for millennia, most notably through agriculture and forestry, breeding, and fermentation of foods and beverages. However, it wasn’t until the turn of the 21st century that we developed a formal engineering discipline around biology^2–4^. There are parallels here with mechanical engineering and thermodynamics. Mechanical systems had been built for thousands of years. Still, it was only during the Industrial Revolution that mechanical engineering became a fully-fledged discipline, predating and driving the scientific development of thermodynamics upon which it now rests^5,6^. Biological engineering today is in a similar position to where mechanical engineering was then, lacking the knowledge and models needed for more predictable design.

Over the last two decades, synthetic biology has made notable progress towards becoming a mature engineering discipline. However, we have also been humbled by how difficult it is to engineer biology. We are still limited to building relatively modest systems, mostly in model organisms, and success is far from guaranteed. This is partly due to technical challenges and our incomplete knowledge of biology. More fundamentally though, it stems from the complex nature of biosystems. Bioengineers deal with *living* systems with long evolutionary histories that grow, display agency, and have potential evolutionary futures. Existing bioengineering paradigms that do not acknowledge this fact will always hold back our ability to engineer biology, regardless of how advanced our technology and biological knowledge may become. We, therefore, need a new kind of engineering that leans on different philosophical assumptions, where change, uncertainty, emergence, and complexity are built in.

A common trend across the field has been to apply classical engineering principles such as standardisation^7–11^, decoupling^12–17^ and abstraction^18,19^ in an attempt to tame biological complexity. However, there has been a conspicuous lack of consideration as to *how* these principles should be applied and whether the fact that biology is an evolved and evolving substrate warrants a different approach. To consider more deeply how engineering principles and the design process should be applied to biology, we must first answer some fundamental questions about engineering and design. What precisely do we do when we engineer something? What is the design process, and how does it work? How does applying engineering principles for a given discipline relate to the substrate it engineers? We need to apply an engineering mindsight to the design of the engineering discipline itself.

### Biological engineering is meta-engineering

By evolving, biosystems produce and refine themselves, and they have a purpose. Just as a hammer is designed for and fulfils the purpose of driving nails, biological objects such as a limb or an enzyme have evolved to perform tasks at which they must succeed (e.g., enabling a crucial chemical conversion). Evolution separates biological systems from non-biological natural systems^20^. Bioengineers do not design simple artefacts; they design systems that are themselves capable of something like design. It allows, and perhaps even requires, that bioengineers take a step back and operate at a higher level of abstraction. We need to become meta-engineers: engineers that engineer the engineering process itself.

An analogy can be made with photography. Before the camera was invented, creativity and skill in image-making were applied directly to the construction of the image itself through marks left on canvas or paper (e.g., where to apply brush strokes and which colours to use). Once the camera was invented, we no longer needed to create the images ourselves. We could instead construct image-making systems. Human skill and creativity were still very much present, but simply applied at a higher level of abstraction (e.g., considering the choice of subject, lens, shutter speed, and a multitude of other parameters). Rather than creating an image directly, the photographer carefully tunes a system and process to create an image the way they want. After the invention of photography, questions about image-making took on a new form. How do we make the best camera? How do we know which settings to use to achieve a desired result? What are the principles and rules for this new image-making art form?^21^

### Engineering design as an evolutionary process

**Fig. 1:**
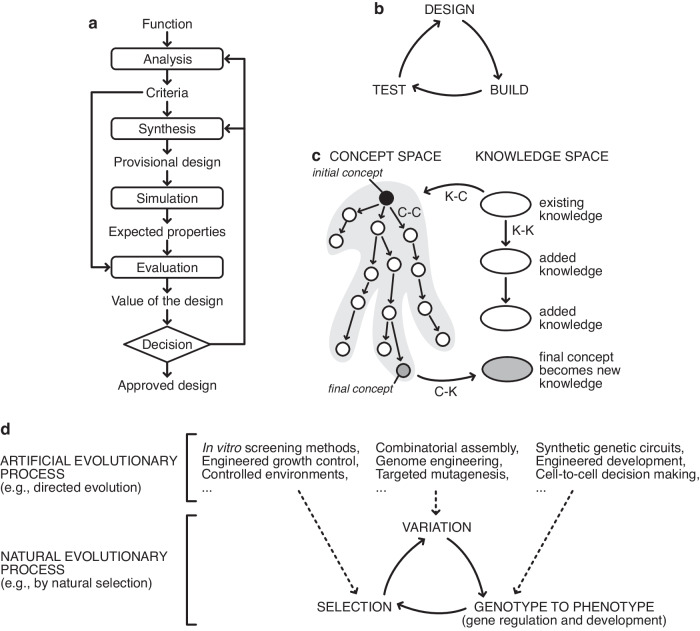
Schematics of various models of the design process and links to natural and artificial evolution. **a** The basic design cycle of Roozenberg and Eekels^9^. **b** The design-build-test cycle that is often referred to in synthetic biology. **c** In CK theory by Hatchuel and Weil^8^, design is a form of knowledge generation that takes place via various operations from concept space to knowledge space, in a cyclical process. **d** Connections between natural evolutionary processes (bottom) and artificial evolutionary processes (top). While natural evolutionary processes harness the innate capabilities of biological populations to evolve through variation, their genotype to phenotype map, and selection, artificial evolutionary processes can modify these core elements using synthetic biology and other engineering tools. This enables the intent of the biological engineer to be embedded within the evolutionary process itself, even though natural evolution has no intent of its own.

The most commonly taught framework for the design process is the German systematic method, which describes design as occurring in three stages: (i) functional, (ii) conceptual, and (iii) embodiment^22^. However, anyone who has carried out even a simple design project knows that it does not follow this simple sequential process. The three stages mingle and overlap and are cycled through many times (Fig. 1a). The design-build-test cycle (Fig. 1b), first proposed by management theorist Kim B. Clark^23^, is popular amongst synthetic biologists and captures the iterative nature of the engineering method more closely. Still, the details of how each stage works and relates to each other are not always well defined. The idea that design is a process that needs no explanation is simply assumed.

In perhaps the most theoretically rigorous attempt at explaining the design process, Armand Hatchuel and Benoît Weil’s CK theory describes design as existing in two spaces: concept space, and knowledge space (Fig. 1c)^24^. Design progresses by translating *concepts*, C (design ideas whose viability are unknown), into *knowledge*, K, in an iterative process consisting of four “operators” collectively named the design square. Concepts can generate new concepts through ideation (C-C operator), concepts can be converted into knowledge through prototyping (C-K operator), knowledge can feed new ideas (K-C operator), and knowledge can be expanded, e.g., through research (K-K operator). By separating the spaces based on logical status, CK theory can incorporate creativity via exploring the concept space while simultaneously expanding and incorporating domain knowledge.

Historian Walter Vincenti’s book ‘What engineers know and how they know it’, is to our knowledge the only thorough attempt at placing the engineering process on a firm epistemological footing^25^. He uses a wealth of case studies from aviation to demonstrate that engineering is its own form of knowledge generation and not simply applied science, concluding that the design process takes place through a cyclic process of idea generation and “vicarious testing”^25^.

All these descriptions of the design process have a common core. They describe design as a cyclic iterative process where multiple concepts or ideas are either modified or recombined, and then produced physically (i.e., a prototype developed) or virtually through simulation or imagination to form candidate solutions that can be tested. The utility (ability to solve the design problem) of these variants is then assessed, and the best candidates are taken forward for further rounds of iteration. This process is directly analogous to biological evolution, where information about variant solutions is encoded in DNA as genotypes, expressed in the physical world via gene expression and development to produce phenotypes, and tested in the environment (Fig. 1d). Sufficiently functional solutions are then taken forward for future rounds of iteration by natural selection. It is therefore not surprising that biological evolution has successfully been applied to solve difficult engineering problems, both in silico with genetic algorithms commonly used to generate design solutions (e.g., novel satellite antenna shapes^26^) and in the lab where directed evolution is widely used to develop and improve enzymes^27^. Biological evolution is a powerful design tool because biological evolution and design follow the same process.

More broadly, evolution is further evident at the macro scale of the entire technosphere. Technologies have been shown to progress through the modification and recombination of existing technologies that are then selected for by free market economics^28^. Macro-evolutionary trends are also apparent in technology just as they are in biology: new “species” of technology can arise, technologies become extinct, and all technologies can ultimately be traced back through a lineage that starts with the first prehistoric tools^29^.

### The evolutionary design spectrum

We propose that all design approaches can be considered evolutionary: they combine some form of variation and selection over many iterations. This allows them to be characterised by the number of variants (the population size) that can be tested simultaneously (*throughput*) and how many design cycles/generations are needed to find a feasible solution (*time*). The product of these two numbers is the total number of variants that can be tested and is the *exploratory power* of the design approach. This will always pale in significance compared to the vast design space for any biological system more complex than a short peptide. Despite this tiny sampling, solutions can still be found in the design space for two reasons: (i) *exploration* – design approaches learn from previous iterations as they go, and (ii) *exploitation* – design approaches are constrained and guided by prior knowledge.

These two forms of learning vastly reduce the exploratory power needed for a design approach to find feasible solutions. Exploration is equivalent to the search performed by natural evolution as it roams the fitness landscape. Natural systems also exploit prior knowledge in the form of the unbroken lineage of their past; every generation of which has proven to be both heritable and adaptive. Exploitation is therefore linked to the evolution of evolvability^30^. Through evolvability, biosystems exploit past evolution in developing body plans, symmetries, functional modules, and other mechanisms that constrain and bias evolution to increase the likelihood of adaptive change. Furthermore, mechanisms, such as learning and epigenetics, which pass information from one individual to the next, are of great importance in evolution^31,32^. Leveraging either form of knowledge decreases the exploratory power needed by the design process and, thus, the time and throughput required to reach performant solutions. Different design processes leverage exploration and exploitation to different extents and have different practical limits on throughput and time. Though natural evolutionary processes have no *intent* (i.e., long-term goal), they do have a *purpose* to increase fitness. This is well illustrated by the Luria-Delbrück experiment, which suggested that adaptation is not due to deliberate genetic mutations in response to selective pressures but instead arises by selecting for random mutations already present in the evolving population^33^. Evolution by natural selection, therefore, lacks the intent we would typically ascribe to the concept of design. However, in artificial evolutionary systems (e.g. directed evolution), the bioengineer can steer the underlying processes toward an intended goal because they control how variation and selection occur, as well as the genotype to phenotype map (Fig. 1d). They can indirectly install their intent through design of the evolutionary process itself.

**Fig. 2:**
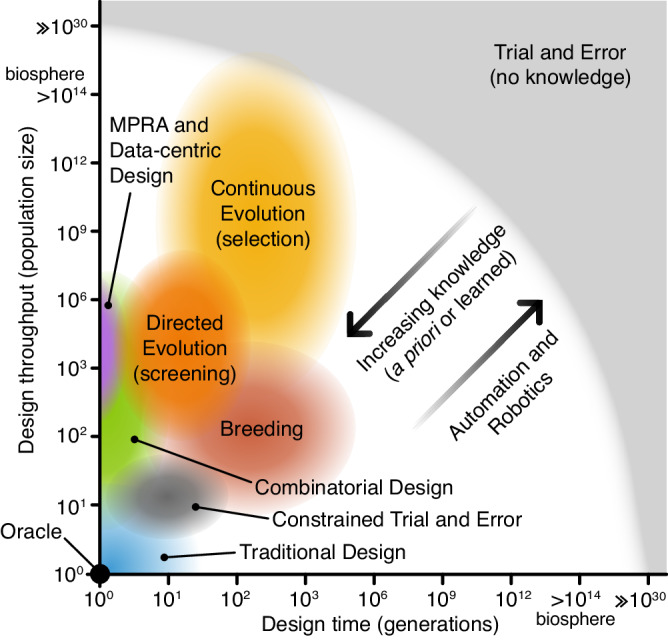
The evolutionary design spectrum. Design approaches in the bottom left have low numbers of variants (population sizes) and design cycles (generations). These require significant prior knowledge for successful design with an oracle able to create a perfect design in a single attempt. Design approaches at the top right make little use of prior knowledge and learning, require less evolvability, and their search is less constrained. Trial and error (with no prior knowledge), falls at this extreme, where hyper-astronomical scales are required to design non-trivial systems. Between these extremes, you have all other design methods. In all these cases, prior knowledge can be used to constrain the design space and learned knowledge can dynamically guide the process on the fly. Furthermore, automation and robotics can help to enhance evolutionary design processes, increasing their design throughput and the number of generations that can be feasibly run.

Any design methodology can therefore be considered a point on a two-dimensional evolutionary design spectrum (Fig. 2) where one dimension is the throughput of the methodology (i.e., how many designs can be created and tested at once) and the other is generation count (i.e., the number of iterations, cycles, generations, that the design process is made up of). Methodologies with high throughputs and high numbers of design cycles have high exploratory power. Conversely, methodologies with lower throughputs and design cycles have lower power and require greater exploitation of prior knowledge and greater evolvability to produce successful designs.

A design methodology must have sufficient specific evolvability to generate phenotypic functions that meet design requirements within the practical constraints of resources and time. This means that for design success, efforts must be made to either maximise evolvability or exploratory power (or both). To improve specific evolvability, exploitation of prior knowledge, ad-hoc exploration, and engineering principles like modularity and hierarchy can be used to constrain the evolutionary landscape to improve the efficiency of search. Take, for example, the simple design problem of creating a synthetic promoter with a given specification (e.g., a desired transcription initiation rate). This could be designed using a variety of methods:

- ***Trial and error*** (hyper-astronomical population size and number of design cycles): Our starting material is a pool of random sequences, there is no prior knowledge about promoters, and no knowledge is carried from one generation to the next. Finding the optimal design requires random trial and error through the entire search space. Even for this small genetic part, the design space is in the order of 10^60^. We would therefore need hyper-astronomical population sizes and timescales for all but the simplest systems. However, if we instead merely need to find a minimally satisfactory solution, there are some cases where a design space is sufficiently populated for this strategy to work. For example, 10% of randomly selected DNA sequences of length 100 nucleotides have been shown to display some promoter activity^34^.
- ***Constrained trial and error*** (moderate population size and number of design cycles): When employing a trial-and-error type approach, it is common for significant amounts of prior knowledge about the system to be used to cut down the search space to a size that can be reasonably assessed through a feasible number of design cycles. In promoter design, this would relate to a core template sequence that is known to have some basal level of activity, with design variants exploring mutations in a small region known to affect function (e.g., RNA polymerase binding affinity). While this makes trial and error a feasible approach, the diversity of the designs is highly limited and knowledge from one design cycle does not help inform the next.
- ***Continuous evolution with selection*** (large population size, large number of design cycles): If a way of coupling utility to survival can be found, e.g., by placing the promoter design candidates upstream of an essential gene in the chosen organism, then continuous evolution is possible^35–37^. This means population size is limited by what can be cultured, not what can be screened, and generation counts are limited by how long the experiment can be run. Thus, hundreds or even thousands of generations are feasible for fast-growing organisms like *Escherichia coli* ^38^.
- ***Directed evolution via screening*** (moderate population size, moderate number of design cycles): Alternatively, we could use prior knowledge to select an imperfect promoter as a starting point and learn from each generation by choosing improved candidates. For example, the promoter can be coupled to a fluorescent reporter gene and screened via cytometry^39^. This way, we could find satisfactory designs with moderate population sizes (e.g., hundreds or thousands).
- ***Massively parallel reporter assays*** (moderate/large population size, single design cycle): Special cases of evolutionary design methods are those with a generation count of one and often a highly constrained design space. Though no continuous learning is possible, design solutions can be found if prior knowledge of the systems can be combined with sufficient throughput. So-called massively parallel reporter assays (MPRAs) often involve fluorescence-activated cell sorting (FACS) and DNA sequencing to reconstruct the evolutionary landscape. In this example, a library of potential promoter designs could be used to drive the expression of a fluorescence reporter gene^40^. Other forms of MPRA use barcoded RNA sequencing to measure transcription rates of variants in a library directly^41^. In principle, these approaches can be expanded into multiple generations by repeating the process, though this may be costly and labour-intensive.
- ***Combinatorial design*** (small/moderate population size, single design cycle): When designing larger or more complex systems, working at single nucleotide resolution may be inefficient. Prior knowledge and abstraction can be used to work with genetic units larger than single nucleotides. For example, if various domains of a promoter are known (e.g., transcription factor binding domains, sigma factor binding domains, TATA box), then these domains can be abstracted into parts that can serve as “genetic units” to be assembled in a combinatorial fashion and the resultant library screened for the best candidates. This strategy depends on abstraction to create complex forms of genetic recombination that allow for search to take place in a highly constrained design space.
- ***Traditional design*** (small population size, small number of design cycles): The traditional design process leverages prior knowledge of the design problem built up through many generations – both formal codified knowledge (e.g., models), tacit knowledge, and intuition to constrain the design space. Design therefore takes place needing only small numbers of variant concepts and iterations. For example, a traditional or rational design approach may involve using existing knowledge of natural functional elements within promoters and mathematical modelling to construct a synthetic promoter that may be iterated through multiple rounds of testing and improvement^42^.
- ***Data-centric design*** (small to large population size, small number of design cycles): The data-centric design approach can be seen as a variant of the traditional design that often employs high-throughput assays (e.g., MPRAs). Typically, a predictive model is initially trained on existing data sets to estimate how well a new variant is expected to work. Models range from a basic linear regression to more complex artificial neural networks that, for the promoter example, would take a DNA sequence as input and return the expected promoter strength. Datasets used for training could contain naturally occurring variants or synthetically designed and synthesised variants that have been characterised in the lab. If searching for a specific promoter strength, the model can also be used to suggest variants that are known to improve the model (e.g., exploring novel areas of design space) in a process called active learning^43^. This type of design process is often coupled with Design of Experiment (DoE) or Bayesian Optimization methods to optimally select new variants to build and test for subsequent design cycles to maximise the information learnt about the relationship between factors of a design/experiment^44–46^. Furthermore, the application of liquid handling robotics allows for far greater numbers of design cycles at the cost of smaller population sizes^47^.
- ***Oracle*** (population of one, single design cycle): If it were possible to have complete knowledge of a system before beginning the design process, we could select the ideal design without any testing. Such an oracle could perform the design process in a single generation with a population size of one, immediately producing an optimal promoter base-for-base.

### Choosing a design approach

As outlined above, different design methods have different practical limits for population size and generation count. This means they leverage exploration and exploitation to differing extents and thus have varying degrees of exploratory power. In addition, there are practical limits to population size and generation count depending on the type of system being designed and the nature of the design problem. For example, a methodology for designing a complex biosystem such as a multi-component genetic circuit may have limited exploratory power due to practical constraints on throughput and time. Conversely, a simple system such as a small protein whose function can be selected for with a continuous growth assay may be carried out in a large population for many generations quite readily. The design process chosen must, therefore, match the constraints of the design problem. As exploratory power reduces, specific evolvability must increase. This can be done through leveraging prior knowledge or applying known engineering principles. However, there may be other ways of improving specific evolvability for specific designs problems, such as by engineering genetic variation^48^.

If evolutionary design approaches are to be used to their full potential for engineering biology, methods to generate and characterise libraries of greater genetic diversity will need to be developed. These libraries should have structural and functional variety and go beyond nucleotide variation or the simple interchanging of parts. What form of synthetic variation is most important and how artificial genetic units should be engineered and selected to maximise the evolvability of resultant populations depends on the size and complexity of the genetic system to be evolved and on technical limitations in terms of generating and reproducing populations, and measuring, screening or selecting the functions of individual genotypes. Different types of genetic systems across scales require different evolutionary design approaches, falling into different regions of the evolutionary design spectrum.

Generally, a more complex biosystem will have greater technical limits on population size and generation times. Therefore, as the complexity of a biosystem increases, the appropriate evolutionary design approach shifts to a point between traditional design and trial and error in the evolutionary design spectrum. Complex systems are difficult for the human mind to design via traditional approaches (bottom left, Fig. 2), but useful results are also unlikely to occur if trial and error is used due to the vastness of the design landscape. The application of deep learning approaches that can exploit high-dimensional gradients inferred from data to guide a design process (i.e., data-centric design) may be able to help in these situations.

In the following sections, we look at how the design methodologies described above can be applied in different ways to engineer biological systems across scales.

### Designing genetic regulatory elements and proteins

Regulatory elements like promoters, ribosome binding sites (RBSs) and terminators have relatively small genotypes making them amenable to direct oligo synthesis or nucleotide mutagenesis of a starting template via error-prone PCR. Activity levels can be coupled to fluorescence to enable selection via FACS^49^, or expression of an antibiotic resistance gene^50^ or DNA polymerase^51^ to affect the reproductive success of the underlying design. This allows for enormous population sizes, where even a single generation may yield good results. Nanopore-based direct-RNA sequencing has also been used to monitor the performance of thousands of transcriptional regulators effectively from large mixed pools of diverse combinatorically assembled designs in a single experiment^52^. Beyond screening, there has been some success in exploiting our knowledge of the modular structure of these biological parts and key motifs within them (e.g., the crucial role of –10 and –35 sites in *Escherichia coli* promoters). However, in virtually all examples to date, current models cannot accurately predict the phenotype from genotype, resulting in the need for nucleotide-level mutations of moderately performant designs to achieve a desired functionality^53,54^.

Proteins have been extensively designed using directed evolution. Sequence lengths range from peptides of a few amino acids, which can potentially be explored exhaustively^55^, but are typically on the order of ~1–3 kb long when encoded in DNA. At this scale, single nucleotide mutations are an effective design strategy, especially if focused on specific target domains or regions. One of the most widely used in vitro methodologies for this task is phage-assisted continuous evolution (PACE)^37^, which enables rapid cycles of evolution by having the gene of interest encoded within a bacteriophage and linking its activity to the fitness of phage replication. Continuous in vivo evolution platforms like OrthoRep^56^ or eMutaT7^57^ are also ideally suited to this task. However, for complex, multi-domain proteins, the addition of structured recombination considering domain boundaries can significantly improve evolvability, as beneficial mutations across different modules can be effectively combined^58,59^. Screening can be performed using in vitro methods with large populations^60^ and a handful of design cycles are typically sufficient to find improved designs.

There is also growing interest in leveraging the power of automation to increase throughput and, thus, exploratory power^61^. Integration of advanced liquid handling robotics and high-throughput assays with existing continuous directed evolution platforms like PACE offers the ability to greatly expand the number of evolutionary trajectories that can be simultaneously explored^62–64^. These provide valuable information regarding the reproducibility of these design processes and help to improve overall reliability. The use of feedback control within these systems can also help to accelerate evolution by reshaping fitness landscapes dynamically over time to support effective adaption of the population^62^.

Outside the lab, emerging machine learning methods for protein structure prediction allow for more traditional and data-centric design approaches to be applied. These can supplement, or in some simple cases, surpass the need for further directed evolution^65–67^. However, designing complex molecular dynamics and unstructured regions remains challenging for computational models. In these cases, computationally efficient coarse-grained models able to capture qualitative features of protein dynamics can be used to provide semi-functional starting points^68^, and high-throughput automated directed evolution applied for further refinement of the designs.

### Designing multi-gene circuits and pathways

Genetic circuits and metabolic pathways are often composed of numerous proteins and regulatory elements that together enable more complex functionalities. Apart from the DNA encoding them being much longer (typically tens of kilobases long), their multi-component structure means that hierarchy begins to play a role. Specifically, variation can occur at the nucleotide level, or modifications can be made at higher levels of organisation (e.g., the swapping of a transcription factor or entire promoter) enabling larger jumps in the genotype space.

To engineer genetic circuits, the most common approach to date has been through model-guided design to find suitable regulatory topologies that implement a desired logic, and then the application of trial and error to vary the components used and introduction of mutations in key regulatory elements to finally tune gene expression levels such that an overall function is optimised. Introducing insulating elements like self-cleaving ribozymes into sub-circuits (e.g., simple logic gates) has enabled traditional design approaches to push forward the complexity of the circuits we can build. However, limits are soon reached, making the design of functional genetic circuits composed of more than ten regulators a challenge. A further difficulty in employing approaches like directed or continuous evolution is the multi-state nature of these systems, where multiple inputs and outputs exist. Unlike simple functional screens that can select designs based on a single output measurement (e.g., fluorescence or enzymatic activity), genetic circuits often require a specific input-output response. These are difficult to screen because of the combinatorial explosion in the number of possible states that must be tested as the number of inputs and outputs grows. Some attempts have been made to overcome this for simple cases (e.g., a single input and output) by removing the need to carry out cell sorting and using changes in growth rate followed by deep sequencing^50^, but the population sizes will always be fundamentally limited, highlighting the need for traditional model-guided or data-centric design approaches in this area.

In contrast, the engineering of metabolic pathways often employs a different approach because maximising a single output metabolite is the goal. In this context, the topology of the pathway is typically fixed (i.e., a specific set of chemical conversions is required), making it well suited to a combinatorial design approach. Sets of enzymes for each step in the pathway can be combined in different ways and genetic regulatory parts with varying activity levels are used to create populations of designs where the set of required enzymes are expressed at differing levels. Because screening can be costly to perform in high throughput (although more feasible than for multi-state genetic circuits), the combinatorial approach can be guided by DoE methods to ensure the most information is learnt after each design cycle^45^.

An interesting alternative to combinatorial design for metabolic pathways is the use of in vitro SCRaMbLE (Synthetic Chromosome Rearrangement and Modification by loxP-mediated Evolution). In this context, the genes encoding each enzyme of the pathway are considered as individual units that can be copied, deleted and their orientation within the pathway flipped by the SCRaMbLE system. This allows for the rapid generation of libraries of metabolic pathway variants with diverse expression levels for each unit^69^. SCRaMbLE could also be applied to gene regulatory networks. However, the enormous design space for most genetic circuits (far exceeding those of metabolic pathways) means that the random SCRaMbLE-based search may not always be effective unless further constrained.

### Designing whole cells

Attempting to design entire cells is yet another step up in terms of complexity, with the need to simultaneously coordinate many different cellular processes to maintain viability and fitness. In this setting, a few mechanistic ‘whole cell’ models of well-studied organisms have been built that recapitulate general cellular phenotypes and can be used to support traditional model-based design approaches at this scale^70–72^. These models have guided host modifications for improved metabolic engineering^73^ and even been used to generate minimised genomes in silico^74^. While these models are sometimes useful, their accuracy is severely lacking due to our limited knowledge of how cells work and missing parameters. It therefore remains to be seen how well predictions from these models generalise outside of the conditions in which they were built. The difficulty in building such models also highlights the need to embrace evolutionary design approaches that rely less on prior knowledge and actively search a design space for desired features.

Beyond computational approaches, long-term experimental evolution studies have shown that novel traits can emerge through natural variation and selection. However, the timescales for discovering these traits can be incredibly slow, requiring thousands of generations^75^. The emerging field of synthetic genomics^76^ offers routes to accelerate this process and overcome our lack of knowledge when designing at a cellular scale. Efforts to synthesise and refactor entire genomes opens opportunities to unravel the core principles underlying genome structure and operation^77–79^. It also allows for the creation of genomes with new capabilities. For example, the Sc2.0 project is resynthesizing and refactoring the *Saccharomyces cerevisiae* genome to extract and consolidate typically unstable elements (e.g., tRNAs^80^), and has introduced genome-wide modifications to enable SCRaMbLE’ing of genome architecture for accelerated in vivo generation of genomic diversity^81^. This system has already demonstrated its ability to evolve strains optimised to produce desired chemicals^82^ and has provided insights into the incredible plasticity of the *S. cerevisiae* genome^83^.

### Designing microbial communities and ecosystems

Many biological processes, such as the nitrogen cycle, arise via the collective action of multiple species. Microbial communities are composed of many different types of organisms that compete, cooperate and tolerate each other. Microbiome engineering^84^ is concerned with finding the community that can perform a desired function, such as degrading a pollutant^85^, increasing digestive health/capabilities^86,87^ or making smart materials^88^ and developing methods to stabilise its existence within the broader environment in which it will be deployed (e.g., the human gut).

Artificial selection (a form of directed evolution) can be used to evolve communities directly^89^. The selection takes place at the population level rather than the individual organism. Because it works with a population of populations, the collective is often referred to as a meta-population – an important concept in ecology^90,91^. Population selection was pioneered in selecting microbial communities in the soil to increase plant biomass^89^ and has since been combined with mathematical models to guide the selection of promising co-cultures^92,93^. A challenge with the approach is that while microorganisms are, in principle, free-living and can be combined at will, this does not mean there is an incentive for functional associations to form. Another issue is handling the dynamics of the communities. For example, when a contributing species goes extinct or fast-growing organisms take over^94^.

To enable the engineering of stable communities, autotrophic dependencies can be exploited and engineered^95^ or other forms of dependency and interaction used^96,97^. In this context, traditional model-based design approaches have proven useful in sufficiently capturing the dynamics of the well-defined communities built in the lab. Deploying these communities into real-world environments, though, remains a significant challenge due to a lack of knowledge regarding the interactions present within complex and diverse native communities that might impact the function of engineered communities and their ability to stably associate. To overcome this, physical separation via encapsulation of engineered microbes has been used to lessen competitive effects and safeguard against unwanted release^98,99^. Looking forward, a mixture of traditional design and evolutionary approaches will likely be required to develop sufficiently robust systems to handle the complexity of real-world microbial communities and ecosystems.

### Towards the effective design of engineered biology

Engineering biology is vastly different from other engineering disciplines. This is not only because living matter has the inherent capacity to evolve but also because human-built “machines” (e.g., cars, computers, etc.) are typically organised differently than biological systems^100^. Both machines and biological organisms contain hierarchical levels. However, machines are designed so that every level is predictable and can be understood independently from other levels. For example, formulating rocket fuel requires distinct expertise from designing a rocket’s navigation system, and while both are required to build a rocket, they can be considered independent during design. In contrast, living systems have organisational levels that are highly interconnected, allowing small mutations to affect broad phenotypes in complex ways. Furthermore, it is common for higher levels or organisation to influence lower ones via downward causation, and human-built machines typically show a hardware-software duality, whereas biological components have blurred lines of information storage and processing (e.g., RNA being a genetic information carrier and a catalytic entity in its own right)^101^. Traditional design works with blueprints, which are typically a one-to-one mapping to the artefact built, while living systems have highly non-linear genotype-phenotype maps that are executed via developmental processes and influenced by environmental factors. These properties mean that engineering frameworks based on reductionism are challenging to apply^102^. In contrast, evolution is naturally adept at handling these properties because it acts holistically on the system within its wider environment.

These difficulties mean that adopting a single design methodology for all bioengineering tasks is likely impossible. Instead, combining more traditional design cycles that use model-based design with high levels of abstraction, may allow a targeted population of designs to be generated upon which more classical evolutionary design approaches can be used to tune and refine partially functional or suboptimal designs^103^. Such hybrid methods are already being applied to minimal genome design, helping to accelerate the generation of a minimal cell with reasonable fitness^104^. Beyond combining design methods, installing new abilities for cells to design themselves through guided mutations^57^ and rearrangements^105^ of their genetic material could further accelerate the design process (e.g., systems like SCRaMbLE). This would work with biology’s innate capacity to evolve, while also allowing us to constrain the paths evolution can take such that prior knowledge can bolster the overall exploratory power of the system. Living systems also tend to evolve to become more evolvable, e.g., by making them more robust or modular^106^. For example, certain mutations can stabilize a protein and make it more susceptible to beneficial mutations^107,108^. Hence, it may be possible to engineer such systems so that they are easier to improve.

Another fundamental challenge that is typical when engineering biology is the definition of a successful design. In most engineering fields, the goal is to maximise some desired utility function. However, with biology, there is always a trade-off in the *utility* of the design (i.e., how well it performs the task we require) and the *fitness* of the resultant biological system (i.e., its reproductive rate). We previously developed the ‘evotype’ as a framework for capturing the evolutionary capacity of a biological design and explicitly highlighting this trade-off^48^. Stated differently, when designing with living matter, we do not only have to consider how well it works, but we also need to consider how it will adapt and evolve during use. Note that this concept integrates the top-down objective (intent) of the bioengineer in the utility with the bottom-up evolutionary aspects captured by fitness.

While we can never fully escape evolution when designing and deploying engineered biological systems, its impact can sometimes be heavily reduced. Recent studies have demonstrated that cooperative assembly of transcription factors can confer regulatory specificity and long-term genetic circuit stability^109^, and the development of highly characterised and insulated landing pads in genomes can both increase the predictability of genetic parts and reduce selective pressures to mutate or remove these foreign elements once installed^17^. In a similar way, dynamic regulation of the burden a genetic circuit imposes on a host through feedback control have also proven successful in extending the functional lifetime of synthetic genetic circuitry and enabling improved production yields^110^. This effort to reduce burden mirrors how computer software needs to be optimized to best exploit underlying hardware. For example, mobile apps are often coded to best utilize lower-power processor instructions and minimize continuously running background computations to conserve battery life. However, the burden in these settings manifests differently. While burden in biological systems can lead to mutations that change the functionality of the genetic circuit, the burden of code running on an electronic computer mainly affects performance and resource usage. In living systems, the “software” component influences how the “hardware” evolves.

In conclusion, evolutionary theory is doubly important when engineering biology: not only is the substrate of synthetic biology a result of evolution, engineering itself can be seen as a form of evolution. It turns out that evolution must be understood as the foundation of the engineering method, as well as the creator and driving force of the living substrate itself. Though biological evolution and technological evolution may seem very different, they are fundamentally the same process. Recognition of this opens the potential for a new way of thinking about how to engineer biology and effectively “design” in the context of living systems.
